# Relationship between the severity of emphysematous change in the lung and morbidity after esophagectomy for esophageal cancer: A retrospective study on a novel strategy for risk prediction

**DOI:** 10.1111/1759-7714.15146

**Published:** 2023-12-09

**Authors:** Tomo Horinouchi, Naoya Yoshida, Shinya Shiraishi, Yoshihiro Hara, Chihiro Matsumoto, Tasuku Toihata, Keisuke Kosumi, Kazuto Harada, Kojiro Eto, Katsuhiro Ogawa, Hiroshi Sawayama, Masaaki Iwatsuki, Yoshifumi Baba, Yuji Miyamoto, Hideo Baba

**Affiliations:** ^1^ Department of Gastroenterological Surgery, Graduate School of Medical Sciences Kumamoto University Kumamoto Japan; ^2^ Department of Diagnostic Radiology, Graduate School of Medical Sciences Kumamoto University Kumamoto Japan

**Keywords:** 13‐2: clinical‐malignant < 13: esophagus, COPD, emphysema, esophageal cancer, esophagectomy, morbidity

## Abstract

**Background:**

Chronic obstructive pulmonary disease (COPD) prevalence increases post‐esophagectomy morbidity. However, the association between COPD severity and post‐esophagectomy morbidity remains unclear because of the lack of an objective method to classify COPD severity. Low attenuation volume ratio (LAVR) estimated using Ziostation2 may reflect the extent of emphysematous changes in the lungs and COPD severity, thereby predicting post‐esophagectomy morbidity.

**Methods:**

A total of 776 patients who underwent curative McKeown esophagectomy for esophageal cancer between April 2005 and June 2021 were included. The patients were divided into high and low preoperative LAVR groups. Short‐term outcomes between the groups were compared for patients who underwent open esophagectomy (OE) and minimally invasive esophagectomy (MIE).

**Results:**

A total of 219 (28%) patients were classified into the high LAVR group. High LAVR was significantly associated with disadvantageous patient characteristics such as advanced age, heavy smoking, and impaired respiratory function. Patients with high LAVR had a significantly higher incidence of severe morbidity and pneumonia after OE. High LAVR was an independent risk factor for severe morbidity (odds ratio [OR], 2.52; 95% confidence interval [CI]: 1.237–5.143; *p* = 0.011) and pneumonia (OR, 2.12; 95% CI: 1.003–4.493; *p* = 0.049) after OE. Meanwhile, LAVR was not correlated with the incidence of post‐MIE morbidity.

**Conclusions:**

LAVR may reflect COPD severity and predict severe morbidity and pneumonia after OE, but not after MIE. Less invasiveness of MIE may alleviate the effects of various disadvantageous backgrounds associated with high LAVR on worse short‐term outcomes.

## Graphical abstract

This study retrospectively investigated the association between the degree of emphysematous changes in the lungs calculated by Ziostation2 and post‐esophagectomy morbidity. High low‐attenuation volume ratio is a novel independent predictor for morbidities after open esophagectomy. Meanwhile, minimally invasive esophagectomy reduces the impact of emphysema severity and ameliorates short‐term patient outcomes.

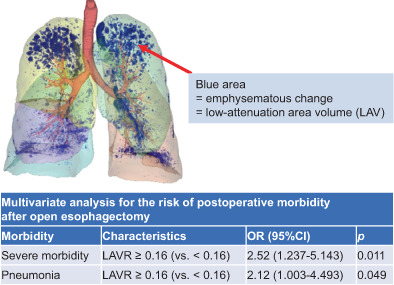

## INTRODUCTION

Esophagectomy for esophageal cancer is a highly invasive surgical procedure associated with frequent postoperative morbidity.^1^ Notably, patients with esophageal cancer frequently smoke and subsequently develop smoking‐related comorbidities, which further increase the risk of post‐esophagectomy morbidity and mortality.^2^, ^3^

Chronic obstructive pulmonary disease (COPD) is strongly correlated with smoking and is often observed in patients with esophageal cancer.^4^ COPD and subsequent impaired respiratory function are reportedly significant risk factors for worse short‐term outcomes after esophagectomy.^5^, ^6^ However, the association between the severity of COPD and the incidence of post‐esophagectomy morbidity has not been investigated because of the lack of a method to objectively quantify COPD severity. The degree of emphysematous change in the lung may serve the estimation of COPD severity and prognosis prediction in patients with COPD.^7^, ^8^ Thus, the quantification of emphysematous change may help investigate the association between COPD severity and post‐esophagectomy morbidity.

**FIGURE 1:**
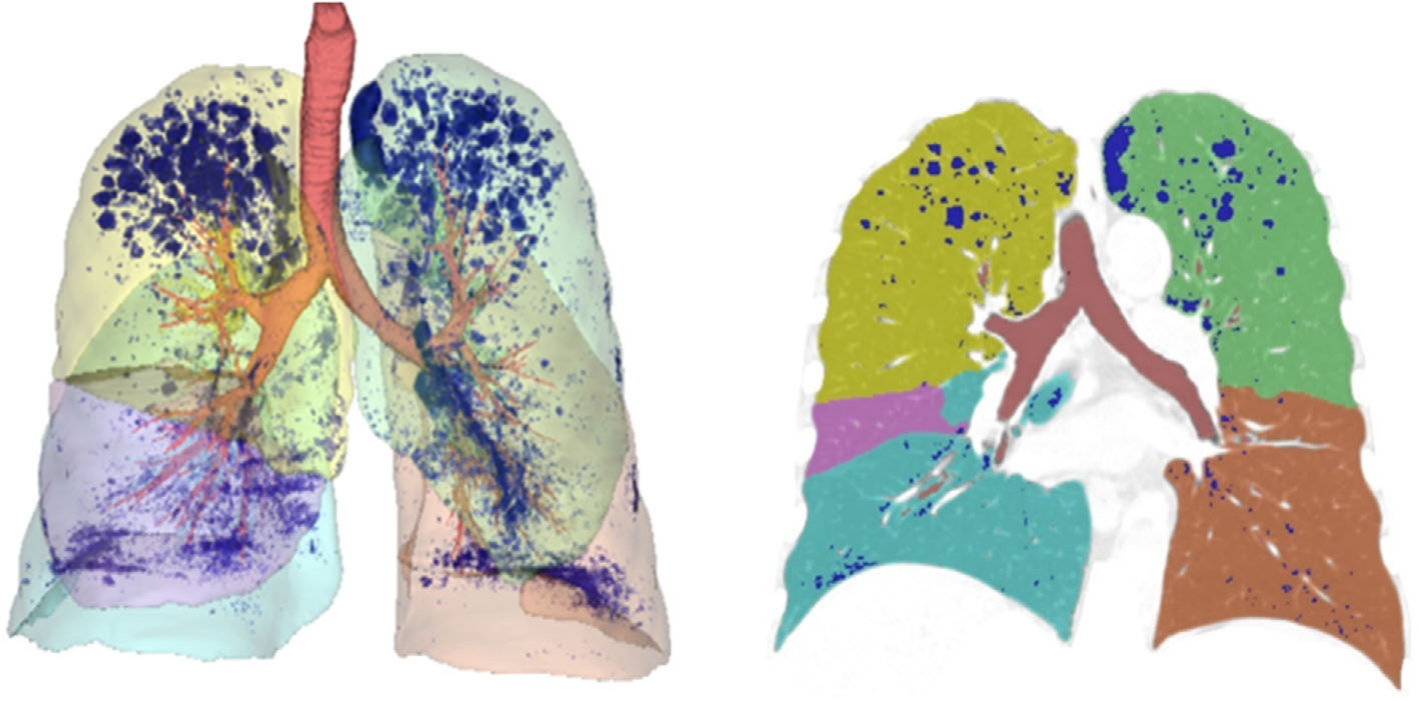
Example of low‐attenuation area in the lung on computed tomography (CT) examined using Ziostation2.

Ziostation2 is a software program used in quantifying emphysematous changes in the lungs on computed tomography (CT). It can automatically calculate the volume of the emphysematous change as a low‐attenuation area volume (LAV) (Figure 1). We hypothesized that the LAV ratio (LAVR) estimated using Ziostation2 can reflect COPD severity, which correlates with post‐esophagectomy morbidity. In this study, we aimed to elucidate whether COPD severity evaluated based on LAVR can predict post‐esophagectomy morbidity.

Furthermore, we investigated whether the impact of COPD severity on the incidence of postoperative morbidity was the same after minimally invasive esophagectomy (MIE) and open esophagectomy (OE). MIE is reportedly less invasive^9^ and predisposes patients to fewer postoperative morbidities than OE.^3^, ^10^ Several studies have suggested that MIE can mitigate the impact of disadvantageous patient characteristics on the incidence of post‐esophagectomy morbidity, thereby improving short‐term outcomes.^11^, ^12^, ^13^ Thus, in this study, we retrospectively investigated the association between COPD severity evaluated using LAVR and postoperative morbidity after OE and MIE.

## METHODS

### Patients

**FIGURE 2:**
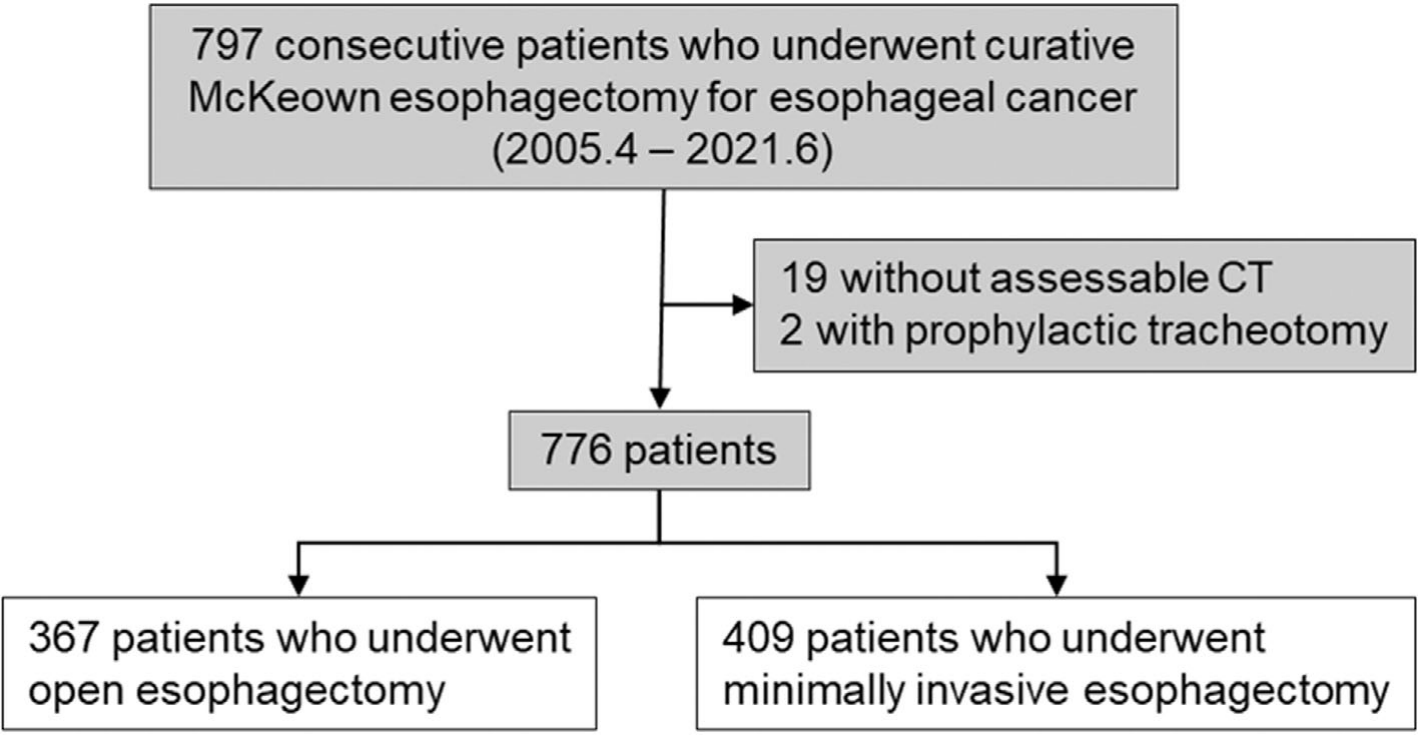
Flow chart of analyzed patients.

A total of 797 consecutive patients underwent curative McKeown esophagectomy for esophageal cancer at Kumamoto University Hospital between April 2005 and June 2021. Among them, 19 patients without assessable CT and 2 patients who underwent prophylactic tracheostomy during esophagectomy were excluded. The association between LAVR and short‐term outcomes after 367 OEs and 409 MIEs was retrospectively compared using an institutional clinical database (Figure 2). The study was conducted in accordance with the ethical standards of the 1975 Declaration of Helsinki. The institutional ethics committee approved all research procedures and waived the need for written informed consent owing to the retrospective nature of the study (registration no. 1909).

### Treatment strategy

Generally, solo esophagectomy without preoperative treatment was performed for non‐T4 tumors without lymph node metastasis on pretreatment diagnosis. Adjuvant (between April 2005 and July 2008) or neoadjuvant (between August 2008 and June 2021) chemotherapy was administered during esophagectomy for non‐T4 tumors with lymph node metastases. Neoadjuvant chemoradiotherapy was administered before esophagectomy in patients with suspected T4 tumor stage. All patients with clinical stage IV tumors had M1 lymph nodes (according to the eighth UICC TNM staging system),^14^ which corresponds to regional lymph nodes (e.g., supraclavicular lymph nodes) in the 11th Japanese Esophageal Cancer Classification,^15^ and underwent neoadjuvant treatment with subsequent esophagectomy. Definitive chemoradiotherapy was administered irrespective of the clinical stage in patients who preferred nonsurgical treatment. Salvage esophagectomy was performed if residual or recurrent tumors were confirmed after definitive chemoradiotherapy.

### Surgery

Three‐incisional McKeown esophagectomy with two or three fields of lymphadenectomy was performed. The extent of lymphadenectomy was determined according to the 2012 guidelines of the Japan Esophageal Society.^16^ MIE was defined thoracoscopic esophagectomy, regardless of the use of laparoscopy, and was performed between May 2011 and June 2021.

### Perioperative management

A 24‐h continuous infusion of a neutrophil elastase inhibitor and a bolus dose of methylprednisolone were administered immediately before esophagectomy. Prophylactic antibiotics were administered intraoperatively every 4 h. Each patient was extubated in the operating room shortly after esophagectomy and treated in the intensive care unit until the first postoperative day. Enteral nutrition was initiated on the first postoperative day.

### Three‐dimensional CT (3D‐CT) reconstruction and measurement of emphysematous changes in the lungs

All CT images were acquired with patients in the supine position and under breath‐holding at full inspiration for esophageal cancer evaluation. CT images of the lung field were reconstructed using 3–5‐mm‐thick slices. Emphysema quantification was performed using a dedicated application on the Ziostation2 (Ziosoft) workstation, with the threshold for the low‐attenuation area (LAA) defined as ≤ − 950 Hounsfield units. Ziostation2 reconstructed 3D‐volume data from lung CT images and automatically extracted data regarding the trachea, bronchus, and lungs, as well as LAA. In addition, LAV and LAVR were calculated using a similar method.

### Definitions of emphysematous change severity on 3D‐CT Volumetry

Emphysematous change severity on CT was determined based on LAVR. The cutoff value of LAVR was determined via receiver‐operating characteristic curve analysis for the forced expiratory volume in 1 second (FEV1) as a percentage of predicted in <80%. Patients were categorized into two groups: high (LAVR ≥0.16%) and low (LAVR <0.16%) groups.

### Definitions of postoperative morbidities

Postoperative morbidity was defined as morbidity with Clavien–Dindo classification (CDc) grade ≥ II.^17^ Severe morbidity was defined as morbidity with CDc grade ≥ IIIb. Pneumonia was defined as the presence of new infiltrates on chest radiography and a positive bronchoalveolar lavage culture.

### Statistical analysis

All statistical analyses were performed using JMP version 14.2 (SAS Institute). Statistical significance was set at a *p*‐value of <0.05. The chi‐square test was performed for comparisons between the high and low LAVR groups. A Mann–Whitney U test was performed to analyze unpaired samples. Logistic regression analysis was performed to determine the odds ratio (OR) and 95% confidence interval (CI) for morbidity. The following variables were used to analyze independent risk factors for severe morbidity and pneumonia: age at esophagectomy (per 10 years), sex (male vs. female), body mass index (BMI) (≥18.5 vs. < 18.5 kg/m^2^), Brinkman index (number of cigarettes/day × smoking duration [year], ≥400 vs. < 400), LAVR (high vs. low), diabetes mellitus (yes vs. no), respiratory disease/COPD (yes vs. no), cardiovascular disease (yes vs. no), American Society of Anesthesiologists physical status (ASA‐PS) (2 and 3 vs. 1), performance status (1 and 2 vs. 0), clinical stage (III and IV vs. 0, I, and II), preoperative treatment (yes vs. no), preoperative radiotherapy (yes vs. no), operative time (for 60 min increases), and blood loss (for 100 g increases). Subsequently, multivariate analysis was performed using selected factors with a *p*‐value of ≤0.1 and recognized variables with a *p*‐value of <0.05 as independent risk factors.

## RESULTS

### Patient characteristics

Table 1 summarizes the patient characteristics according to LAVR. Of all patients, 219 (28%) were classified into the high LAVR group. Compared with low LAVR, high LAVR was significantly correlated with advanced age (*p* = 0.014), lower BMI (*p* < 0.0001), higher Brinkman index (*p* < 0.0001), worse ASA‐PS (*p* = 0.0001), lower FEV1/forced vital capacity (FVC) ratio (*p* < 0.0001), and higher prevalence of respiratory disease and COPD (*p* < 0.0001). A similar tendency was observed when the clinical features of patients who underwent OE and MIE were investigated separately (Tables S1. and S2).

**TABLE 1:** Characteristics of the patients according to the low attenuation volume ratio.

		Low attenuation volume ratio		*p*‐value
Clinical, epidemiological, and surgical feature	Total *N*	High	Low	
All cases	776	219 (28%)	557 (72%)	
Age, mean ± SD	66.2 ± 8.2	67.3 ± 7.9	65.7 ± 8.2	0.014
Sex (male)	672 (87%)	194 (89%)	478 (86%)	0.35
Body mass index, mean ± SD (kg/m^2^)	21.8 ± 3.1	20.6 ± 3.1	22.3 ± 3.0	<0.0001
^a^Brinkman index, mean ± SD	770 ± 570	950 ± 590	690 ± 550	<0.0001
Performance status				0.065
0	685 (88%)	188 (86%)	497 (89%)	
1	81 (11%)	25 (11%)	56 (10%)	
2	10 (1%)	6 (3%)	4 (1%)	
American Society of Anesthesiologists physical status				0.0001
1	157 (20%)	32 (15%)	125 (22%)	
2	573 (74%)	163 (74%)	410 (74%)	
3	46 (6%)	24 (11%)	22 (4%)	
%VC, mean ± SD (%)	102.2 ± 14.8	101.3 ± 14.8	102.5 ± 14.7	0.33
FEV1/FVC ratio, mean ± SD (%)	73.6 ± 8.8	70.3 ± 11.0	74.9 ± 7.5	<0.0001
Comorbidity				
Diabetes mellitus	150 (19%)	38 (17%)	112 (20%)	0.42
Respiratory disease	268 (35%)	114 (52%)	154 (28%)	<0.0001
Chronic obstructive pulmonary disease	233 (30%)	102 (47%)	131 (24%)	<0.0001
Cardiovascular disease	400 (52%)	114 (52%)	286 (51%)	0.87
Clinical stage				0.21
0, I	306 (39%)	74 (34%)	232 (42%)	
II	141 (18%)	46 (21%)	95 (17%)	
III	231 (30%)	71 (32%)	160 (29%)	
IV	98 (13%)	28 (13%)	70 (12%)	
Preoperative treatment				0.15
Absent	414 (53%)	113 (52%)	301 (54%)	
Neoadjuvant chemotherapy	257 (33%)	78 (36%)	179 (32%)	
Neoadjuvant chemoradiotherapy	69 (9%)	23 (10%)	46 (8%)	
Definitive chemoradiotherapy	36 (5%)	5 (2%)	31 (6%)	
Thoracic procedure				0.30
OE	367 (47%)	97 (44%)	270 (48%)	
MIE	409 (53%)	122 (56%)	287 (52%)	

Abbreviations: FEV, forced expiratory volume; FVC, forced vital capacity; MIE, minimally invasive esophagectomy; OE, open esophagectomy; SD, standard deviation; VC, vital capacity.

^a^ Brinkman index was calculated as follows: number of cigarettes/day x smoking duration (year).

### Association between the LAVR and the FEV1/FVC ratio

**FIGURE 3:**
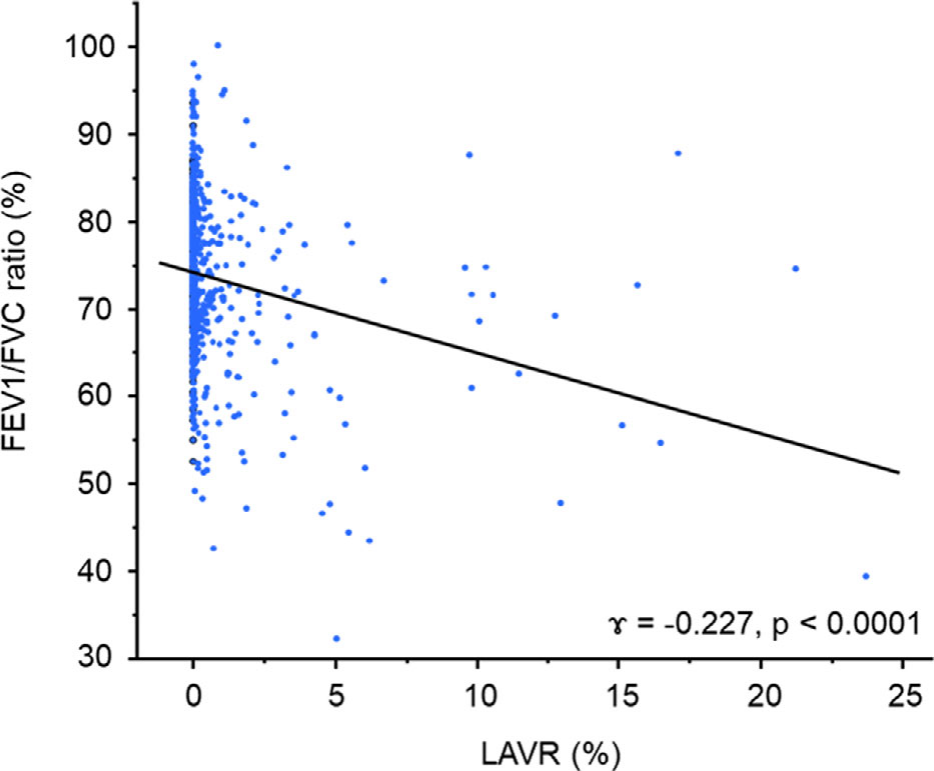
Association between the low‐attenuation volume ratio and the percent forced expiratory volume in 1 s. *FEV*, forced expiratory volume; *LAVR*, low‐attenuation volume ratio.

A weak negative association was observed between LAVR and FEV1/FVC ratio (r = −0.227, *p* < 0.0001) (Figure 3).

### Short‐term OE and MIE outcomes

Table 2 summarizes the short‐term OE outcomes. Compared with low LAVR, high LAVR was significantly correlated with higher frequency of severe morbidity (*p* = 0.010) and pneumonia (*p* = 0.014). However, LAVR was not correlated with the incidence of post‐MIE morbidity (Table 3).

**TABLE 2:** Short‐term outcomes in open esophagectomy.

		Low attenuation volume ratio		*p*‐value
Variables	Total *N*	High	Low	
All cases	367	97	270	
Operative time, mean ± SD (min)	550 ± 120	540 ± 120	550 ± 120	0.39
Bleeding, mean ± SD (g)	660 ± 1000	730 ± 1600	640 ± 650	0.46
Any morbidity, CDc ≥ II	163 (44%)	48 (49%)	115 (43%)	0.28
Severe morbidity, CDc ≥ IIIb	44 (12%)	19 (20%)	25 (9%)	0.010
Pneumonia	35 (10%)	16 (16%)	19 (7%)	0.014
Surgical site infection	102 (28%)	23 (24%)	79 (29%)	0.36
Anastomotic leakage	49 (13%)	11 (11%)	38 (14%)	0.60
Cardiovascular morbidity	18 (5%)	6 (6%)	12 (4%)	0.58

Abbreviations: CDc, Clavien–Dindo classification SD, standard deviation.

**TABLE 3:** Short‐term outcomes in minimally invasive esophagectomy.

		Low attenuation volume ratio		*p*‐value
Variables	Total *N*	High	Low	
All cases	409	122	287	
Operative time, mean ± SD (min)	580 ± 100	570 ± 100	580 ± 100	0.14
Bleeding, mean ± SD (g)	310 ± 1300	230 ± 250	340 ± 1600	0.43
Any morbidity, CDc ≥ II	144 (35%)	47 (39%)	97 (34%)	0.37
Severe morbidity, CDc ≥ IIIb	48 (12%)	14 (10%)	34 (12%)	1.00
Pneumonia	37 (9%)	12 (10%)	25 (9%)	0.71
Surgical site infection	96 (23%)	30 (25%)	66 (23%)	0.80
Anastomotic leakage	53 (13%)	12 (10%)	41 (14%)	0.26
Cardiovascular morbidity	30 (7%)	7 (6%)	23 (8%)	0.54

Abbreviations: CDc, Clavien–Dindo classification; SD, standard deviation.

### Risk factors for severe morbidity and pneumonia after OE

Tables 4 and 5 show the risk factors for severe morbidity and pneumonia after OE, respectively. Multivariate analysis revealed that high LAVR was an independent risk factor for severe morbidity (OR = 2.52; 95% CI: 1.237–5.143; *p* = 0.011), along with cardiovascular disease (OR = 2.62; 95% CI: 1.314–5.245; *p* = 0.0063). Moreover, high LAVR was an independent risk factor for postoperative pneumonia (OR = 2.12; 95% CI: 1.003–4.493; *p* = 0.049).

**TABLE 4:** Logistic regression analysis for severe morbidity in patients who underwent open esophagectomy.

	Univariate analysis		Multivariate analysis	
Characteristics	OR (95% CI)	*p*‐value	OR (95% CI)	*p*‐value
Respiratory disease (vs. no)	1.74 (0.923–3.271)	0.087	1.43 (0.717–2.837)	0.31
Cardiovascular disease (vs. no)	2.50 (1.289–4.833)	0.0067	2.62 (1.314–5.245)	0.0063
Operation time (for 60 min increase)	1.21 (1.054–1.392)	0.0085	1.15 (0.976–1.354)	0.095
Bleeding (for 100 g increase)	1.07 (1.008–1.130)	0.0007	1.06 (0.989–1.129)	0.10
Low attenuation volume ratio ≥0.16 (vs. < 0.16)	2.39 (1.248–4.567)	0.0086	2.52 (1.237–5.143)	0.011

Abbreviations: CI, confidence interval; OR, odds ratio.

**TABLE 5:** Logistic regression analysis for pneumonia in patients who underwent open esophagectomy.

	Univariate analysis		Multivariate analysis	
Characteristics	OR (95% CI)	*p*‐value	OR (95% CI)	*p*‐value
^a^Brinkman index ≥400 (vs. < 400)	2.58 (0.886–7.535)	0.082	2.31 (0.777–6.894)	0.13
Performance status 1 and 2 (vs. 0)	2.26 (1.047–4.875)	0.038	2.06 (0.926–4.570)	0.076
Chronic obstructive pulmonary disease	1.82 (0.893–3.696)	0.099	1.24 (0.583–2.649)	0.57
Low attenuation volume ratio ≥0.16 (vs. < 0.16)	2.61 (1.282–5.311)	0.0082	2.12 (1.003–4.493)	0.049

Abbreviations: CI, confidence interval; OR, odds ratio.

^a^ Brinkman index was calculated as follows: number of cigarettes/day x smoking duration (year).

## DISCUSSION

This study elucidated the association between COPD severity estimated based on LAVR and the short‐term outcomes of curative McKeown esophagectomy for esophageal cancer. First, high LAVR was significantly associated with a higher frequency of COPD and impaired respiratory function, which may reflect COPD severity. Second, high LAVR was significantly associated with several patient features that increased the incidence of postoperative morbidity and mortality, such as advanced age, low BMI, high Brinkman index, and poor ASA‐PS. Third, the incidence of severe postoperative morbidity and pneumonia was significantly higher in patients with high LAVR who underwent OE, and high LAVR was found to be an independent risk factor for such morbidities. Fourth, LAVR was not correlated with the incidence of postoperative morbidities in patients with MIE. To the best of our knowledge, this is the first study to show that COPD severity estimated using LAVR can be a useful predictor of post‐esophagectomy morbidities.

Several studies have suggested that respiratory comorbidities can predispose patients to post‐esophagectomy morbidity.^2^, ^5^, ^6^ However, the association between the severity of such comorbidities and the incidence of post‐esophagectomy morbidity has not yet been elucidated, probably due to the lack of a suitable indicator for objectively assessing comorbidity severity. Spirometry,^18^ CT‐assessed emphysematous changes in the lungs,^7^, ^8^ and the levels of several biomarkers^19^ are possible indicators for estimating the severity of respiratory diseases. However, there are several limitations in the precise assessment of these indicators. Although spirometry is a useful tool for evaluating respiratory function, the results may vary based on the physical condition and cognitive ability of patients, particularly in elderly patients.^20^ Emphysematous changes on CT cannot be quantified without using other equipment. Thus, it is necessary to establish a strategy to objectively and quantitatively assess the severity of respiratory diseases associated with post‐esophagectomy morbidity. Ziostation2 can be used to perform an objective and quantitative evaluation of emphysematous changes, regardless of the physical condition and cognitive ability of patients. Moreover, in the recent viral epidemic, the risk of infection of medical staff may be lower using Ziostation2 than using spirometry.

Ziostation2 can easily calculate LAVR, which is useful to estimate the severity of COPD. In addition, high LAVR reflects the clinical characteristics of patients with esophageal cancer, such as advanced age, low BMI, heavy smoking, poor ASA‐PS, impaired respiratory function, and high prevalence of respiratory disease. Previous studies have suggested that these characteristics are independent risk factors for respiratory morbidity after esophagectomy.^21^, ^22^, ^23^, ^24^, ^25^ LAVR is a surrogate marker of such undesirable patient characteristics; hence, the LAVR is a significant predictor of severe morbidity and pneumonia after OE.

In this study, the association between COPD severity and postoperative morbidities was not confirmed in patients who underwent MIE, implying the superiority of less invasive surgery in terms of better short‐term outcomes.^3^, ^10^, ^26^ Several studies have reported improved short‐term post‐MIE outcomes in patients with disadvantageous characteristics. In a previous study, preoperative malnutrition, estimated using the nutritional status score, was significantly associated with severe, respiratory, and cardiovascular morbidities after OE in patients with esophageal cancer. However, it did not increase the incidence post‐MIE morbidities.^13^ Moreover, high preoperative HbA1c levels constituted a significant risk factor for surgical site infection (SSI) after OE; nevertheless, it did not increase the incidence of post‐MIE SSI.^11^ These studies support the present results that MIE can alleviate the effects of various disadvantageous patient characteristics on the incidence of post‐esophagectomy morbidities.

Based on this study, various prophylactic strategies are required to prevent post‐OE morbidity in patients with high LAVR.^27^ Smoking cessation^4^ and perioperative respiratory rehabilitation^28^ are crucial in respiratory morbidity prevention. Additionally, oral hygiene^29^ and nutritional interventions may help reduce the incidence of respiratory and infectious morbidities.^30^ Moreover, the implementation of enhanced recovery after surgery protocols^31^ and perioperative management by multidisciplinary perioperative teams^32^, ^33^ may be useful. In addition, nonsurgical treatments such as chemoradiotherapy, chemotherapy, immunotherapy may also be considered for high‐risk patients with a considerably high LAVR.

Further minimally invasive surgical procedures may help reduce post‐esophagectomy morbidities in patients with high LAVRs. A randomized controlled trial suggested that robot‐assisted esophagectomy may reduce the incidence of postoperative pneumonia compared with MIE.^34^ One retrospective study in 200 patients with esophageal squamous cell carcinoma reported that mediastinoscopic esophagectomy significantly reduces the incidence of respiratory morbidity compared with MIE.^35^ Post‐esophagectomy morbidity can reduce patient prognosis.^36^ A recent cross‐sectional study in 544 patients with esophageal cancer demonstrated that adequate preoperative prophylaxis may improve both short‐term outcomes and prognosis after esophagectomy.^37^ Overall, various measures are required to improve the survival outcomes in high‐risk patients with postoperative morbidity.

Despite the discrete evaluation and analysis, this study had several limitations. This was a single‐center study with a retrospective design and a long duration; hence, historical biases regarding the treatment strategy, surgical procedure, and perioperative management could not be eliminated. Notably, MIE was performed more recently than OE, which may have affected the incidence of postoperative morbidity. Moreover, the exclusion of 19 patients due to a lack of CT data may have caused a selection bias. Thus, future studies using large multi‐institutional cohorts are necessary to establish the usefulness of LAVR in post‐esophagectomy morbidity assessment.

In conclusion, Ziostation2 can detect severe preoperative emphysematous changes in the lungs based on LAVR, which is a significant predictor of severe morbidity and pneumonia after OE. Furthermore, MIE may reduce the impact of a high preoperative LAVR on short‐term outcomes. Surgeons need to pay attention to the risk of frequent post‐OE morbidities in patients with high LAVRs.

## AUTHOR CONTRIBUTIONS

All authors had full access to the data in the study and take responsibility for the integrity of the data and the accuracy of the data analysis. Conceptualization, N.Y. and H.B.; Data Curation, T.H., N.Y., Y.H., C.M., T.T., K.K., K.H., K.E., K.O., H.S., M.I., Y.B. and Y.M.; Funding Acquisition, N.Y. and H.B.; Project Administration, N.Y. and H.B.; Resources, N.Y. and H.B.; Supervision, N.Y. and H.B.; Visualization, T.H. and N.Y.; Writing – Original Draft Preparation, T.H.; Writing – Review & Editing, N.Y., S.S. and H.B.

## CONFLICT OF INTEREST STATEMENT

All authors have no conflicts of interest to declare.

## Supporting information


**Table S1.** Characteristics of the patients who underwent open esophagectomy based on the low‐attenuation volume ratio.
**Table S2.** Characteristics of the patients who underwent minimally invasive esophagectomy based on the low‐attenuation volume ratio.

## References

[1] Marubashi S , Takahashi A , Kakeji Y , Hasegawa H , Ueno H , Eguchi S , et al. Surgical outcomes in gastroenterological surgery in Japan: report of the National Clinical Database 2011–2019. Ann Gastroenterol Surg. 2021;5:639–658. 34585049 10.1002/ags3.12462 PMC8452469

[2] Klevebro F , Elliott JA , Slaman A , Vermeulen BD , Kamiya S , Rosman C , et al. Cardiorespiratory comorbidity and postoperative complications following esophagectomy: a European multicenter cohort study. Ann Surg Oncol. 2019;26:2864–2873. 31183640 10.1245/s10434-019-07478-6 PMC6682565

[3] Yoshida N , Yamamoto H , Baba H , Miyata H , Watanabe M , Toh Y , et al. Can minimally invasive esophagectomy replace open esophagectomy for esophageal cancer? Latest analysis of 24,233 esophagectomies from the Japanese National Clinical Database. Ann Surg. 2020;272:118–124. 30720501 10.1097/SLA.0000000000003222

[4] Yoshida N , Baba Y , Hiyoshi Y , Shigaki H , Kurashige J , Sakamoto Y , et al. Duration of smoking cessation and postoperative morbidity after esophagectomy for esophageal cancer: how long should patients stop smoking before surgery? World J Surg. 2016;40:142–147. 26330238 10.1007/s00268-015-3236-9

[5] Ohi M , Toiyama Y , Omura Y , Ichikawa T , Yasuda H , Okugawa Y , et al. Risk factors and measures of pulmonary complications after thoracoscopic esophagectomy for esophageal cancer. Surg Today. 2019;49:176–186. 30255330 10.1007/s00595-018-1721-0

[6] Thomas M , Defraene G , Lambrecht M , Deng W , Moons J , Nafteux P , et al. NTCP model for postoperative complications and one‐year mortality after trimodality treatment in oesophageal cancer. Radiother Oncol. 2019;141:33–40. 31630867 10.1016/j.radonc.2019.09.015

[7] Feldhaus FW , Theilig DC , Hubner RH , Kuhnigk JM , Neumann K , Doellinger F . Quantitative CT analysis in patients with pulmonary emphysema: is lung function influenced by concomitant unspecific pulmonary fibrosis? Int J Chron Obstruct Pulmon Dis. 2019;14:1583–1593. 31409984 10.2147/COPD.S204007 PMC6646798

[8] Vimala LR , Gibikote S , Irodi A , Rajan M , Christopher DJ . Correlation of quantitative and qualitative parameters of high‐resolution computed tomography with pulmonary function test for diagnosing and assessing the severity of obstructive pulmonary disease. Pol J Radiol. 2019;84:e381–e388. 31969954 10.5114/pjr.2019.89306 PMC6964351

[9] Kanekiyo S , Takeda S , Tsutsui M , Nishiyama M , Kitahara M , Shindo Y , et al. Low invasiveness of thoracoscopic esophagectomy in the prone position for esophageal cancer: a propensity score‐matched comparison of operative approaches between thoracoscopic and open esophagectomy. Surg Endosc. 2018;32:1945–1953. 29075967 10.1007/s00464-017-5888-z

[10] Biere SS , van Berge Henegouwen MI , Maas KW , et al. Minimally invasive versus open oesophagectomy for patients with oesophageal cancer: a multicentre, open‐label, randomised controlled trial. Lancet. 2012;379:1887–1892. 22552194 10.1016/S0140-6736(12)60516-9

[11] Yamane T , Yoshida N , Horinouchi T , Morinaga T , Eto K , Harada K , et al. Minimally invasive esophagectomy may contribute to low incidence of postoperative surgical site infection in patients with poor glycemic control. Langenbecks Arch Surg. 2022;407:579–585. 34459983 10.1007/s00423-021-02306-6

[12] Yoshida N , Horinouchi T , Toihata T , Harada K , Eto K , Sawayama H , et al. Clinical significance of pretreatment red blood cell distribution width as a predictive marker for postoperative morbidity after esophagectomy for esophageal cancer: a retrospective study. Ann Surg Oncol. 2022;29:606–613. 34467503 10.1245/s10434-021-10719-2 PMC8407934

[13] Horinouchi T , Yoshida N , Harada K , Eto K , Sawayama H , Iwatsuki M , et al. A retrospective study of preoperative malnutrition based on the controlling nutritional status score as an associated marker for short‐term outcomes after open and minimally invasive esophagectomy for esophageal cancer. Langenbecks Arch Surg. 2022;407:3367–3375. 35976434 10.1007/s00423-022-02655-w

[14] Rice TW , Ishwaran H , Ferguson MK , Blackstone EH , Goldstraw P . Cancer of the esophagus and esophagogastric junction: an eighth edition staging primer. J Thorac Oncol. 2017;12:36–42. 27810391 10.1016/j.jtho.2016.10.016 PMC5591443

[15] Japan Esophageal Society . Japanese classification of esophageal cancer, 11th edition: part I. Esophagus. 2017;14:1–36. 28111535 10.1007/s10388-016-0551-7 PMC5222932

[16] Kuwano H , Nishimura Y , Oyama T , Kato H , Kitagawa Y , Kusano M , et al. Guidelines for diagnosis and treatment of carcinoma of the esophagus April 2012 edited by the Japan esophageal society. Esophagus. 2015;12:1–30. 25620903 10.1007/s10388-014-0465-1 PMC4297610

[17] Dindo D , Demartines N , Clavien PA . Classification of surgical complications: a new proposal with evaluation in a cohort of 6336 patients and results of a survey. Ann Surg. 2004;240:205–213. 15273542 10.1097/01.sla.0000133083.54934.ae PMC1360123

[18] Agustí A , Celli BR , Criner GJ, et al. Global initiative for chronic obstructive lung disease 2023 report: GOLD executive summary. Eur Respir J. 2023;61:1–26. 10.1183/13993003.00239-2023 PMC10066569 36858443

[19] Fermont JM , Masconi KL , Jensen MT , Ferrari R , di Lorenzo VAP , Marott JM , et al. Biomarkers and clinical outcomes in COPD: a systematic review and meta‐analysis. Thorax. 2019;74:439–446. 30617161 10.1136/thoraxjnl-2018-211855 PMC6484697

[20] Gu C , Ma M , Xu J , Yuan W , Li R , Guo H , et al. Association between pulmonary ventilatory function and mild cognitive impairment: a population‐based study in rural China. Front Public Health. 2022;10:1038576. 10.3389/fpubh.2022.1038576 PMC9666756 36408049

[21] Murakami K , Akutsu Y , Miyata H , Toh Y , Toyozumi T , Kakeji Y , et al. Essential risk factors for operative mortality in elderly esophageal cancer patients registered in the National Clinical Database of Japan. Esophagus. 2023;20:39–47. 36125625 10.1007/s10388-022-00957-y

[22] Hirano Y , Kaneko H , Konishi T , Itoh H , Matsuda S , Kawakubo H , et al. Impact of body mass index on major complications, multiple complications, in‐hospital mortality, and failure to rescue after esophagectomy for esophageal cancer: a nationwide inpatient database study in Japan. Ann Surg. 2023;277:e785–e792. 35129484 10.1097/SLA.0000000000005321

[23] Uchihara T , Yoshida N , Baba Y , Yagi T , Toihata T , Oda E , et al. Risk factors for pulmonary morbidities after minimally invasive esophagectomy for esophageal cancer. Surg Endosc. 2018;32:2852–2858. 29273870 10.1007/s00464-017-5993-z

[24] Yoshida N , Watanabe M , Baba Y , Iwagami S , Ishimoto T , Iwatsuki M , et al. Risk factors for pulmonary complications after esophagectomy for esophageal cancer. Surg Today. 2014;44:526–532. 23584275 10.1007/s00595-013-0577-6

[25] Sato S , Nakatani E , Higashizono K , Nagai E , Taki Y , Nishida M , et al. The impact of the American Society of Anesthesiology‐Physical Status classification system on the treatment and prognosis of patients with esophageal cancer undergoing esophagectomy. Int J Clin Oncol. 2022;27:1289–1299. 35674969 10.1007/s10147-022-02190-0

[26] Dyas AR , Stuart CM , Bronsert MR , et al. Minimally invasive surgery is associated with decreased postoperative complications after esophagectomy. J Thorac Cardiovasc Surg. 2023;166:268–278. 36577613 10.1016/j.jtcvs.2022.11.026

[27] Yoshida N , Harada K , Iwatsuki M , Baba Y , Baba H . Precautions for avoiding pulmonary morbidity after esophagectomy. Ann Gastroenterol Surg. 2020;4:480–484. 33005841 10.1002/ags3.12354 PMC7511556

[28] Bolger JC , Loughney L , Tully R , Cunningham M , Keogh S , McCaffrey N , et al. Perioperative prehabilitation and rehabilitation in esophagogastric malignancies: a systematic review. Dis Esophagus. 2019;32:doz058. 31206582 10.1093/dote/doz058

[29] Papaconstantinou D , Fournaridi AV , Tasioudi K , Lidoriki I , Michalinos A , Konstantoudakis G , et al. Identifying the role of preoperative oral/dental health care in post‐esophagectomy pulmonary complications: a systematic review and meta‐analysis. Dis Esophagus. 2023;36:doac062. 36097793 10.1093/dote/doac062

[30] Kanekiyo S , Takeda S , Iida M , Nishiyama M , Kitahara M , Shindo Y , et al. Efficacy of perioperative immunonutrition in esophageal cancer patients undergoing esophagectomy. Nutrition. 2019;59:96–102. 30468936 10.1016/j.nut.2018.08.006

[31] Tang Z , Lu M , Qu C , Zhang Y , Li L , Li S , et al. Enhanced recovery after surgery improves short‐term outcomes in patients undergoing esophagectomy. Ann Thorac Surg. 2022;114:1197–1204. 34624264 10.1016/j.athoracsur.2021.08.073

[32] Kawata S , Hiramatsu Y , Shirai Y , Watanabe K , Nagafusa T , Matsumoto T , et al. Multidisciplinary team management for prevention of pneumonia and long‐term weight loss after esophagectomy: a single‐center retrospective study. Esophagus. 2020;17:270–278. 32026048 10.1007/s10388-020-00721-0 PMC7316685

[33] Watanabe M , Mine S , Nishida K , Yamada K , Shigaki H , Oya S , et al. Improvement in short‐term outcomes after esophagectomy with a multidisciplinary perioperative care team. Esophagus. 2016;13:337–342.

[34] Magouliotis DE , Zotos PA , Fergadi MP , Koukousaki D , Zacharoulis D , Diamantis A , et al. Meta‐analysis of robot‐assisted versus video‐assisted McKeown esophagectomy for esophageal cancer. Updates Surg. 2022;74:1501–1510. 35932405 10.1007/s13304-022-01343-0

[35] Shi K , Qian R , Zhang X , Jin Z , Lin T , Lang B , et al. Video‐assisted mediastinoscopic and laparoscopic transhiatal esophagectomy for esophageal cancer. Surg Endosc. 2022;36:4207–4214. 34642798 10.1007/s00464-021-08754-x

[36] Horinouchi T , Yoshida N , Toihata T , Harada K , Eto K , Ogawa K , et al. Postoperative respiratory morbidity can adversely affect prognosis in thoracoscopic esophagectomy for esophageal cancer: a retrospective study. Surg Endosc. 2023;37:2104–2111. 36316584 10.1007/s00464-022-09711-y

[37] Yoshida N , Eto K , Horinouchi T , Harada K , Sawayama H , Ogawa K , et al. Preoperative smoking cessation and prognosis after curative esophagectomy for esophageal cancer: a cross‐sectional study. Ann Surg Oncol. 2022;29:8172–8180. 36029384 10.1245/s10434-022-12433-z

