# Supplementary material for: Relationship between the severity of emphysematous change in the lung and morbidity after esophagectomy for esophageal cancer: A retrospective study on a novel strategy for risk prediction
Source: Thorac Cancer. 2023 Dec 9;15(1):15–22. doi: 10.1111/1759-7714.15146 (PMC10761611; doi:10.1111/1759-7714.15146)
Supplement: Supplementary file 1 — Table S1. Characteristics of the patients who underwent open esophagectomy based on the low‐attenuation volume ratio. Table S2. Characteristics of the patients who underwent minimally invasive esophagectomy based on the low‐attenuation volume ratio. [file TCA-15-15-s001.docx]

Supplementary Digital Content Table 1. Characteristics of the patients who underwent open esophagectomy based on the low-attenuation volume ratio

|  |  | Low attenuation volume ratio | | *p* |
| --- | --- | --- | --- | --- |
| Clinical, epidemiological, and surgical feature | Total *N* | High | Low |  |
| All cases | 367 | 97 | 270 |  |
| Age, mean ± SD | 66.2 ± 8.6 | 67.6 ± 8.3 | 65.7 ± 8.6 | 0.062 |
| Sex Male | 322 (88%) | 87 (90%) | 235 (87%) | 0.59 |
| Body mass index, mean ± SD (kg/m²) | 21.6 ± 3.1 | 20.1 ± 3.1 | 22.1 ± 2.9 | <0.0001 |
| *Brinkman Index, mean ± SD | 790 ± 620 | 970 ± 640 | 730 ± 600 | 0.0008 |
| Performance status |  |  |  | 0.012 |
| 0 | 300 (82%) | 73 (75%) | 227 (84%) |  |
| 1 | 60 (16%) | 19 (20%) | 41 (15%) |  |
| 2 | 7 (2%) | 5 (5%) | 2 (1%) |  |
| American Society of Anesthesiologists Physical Status |  |  |  | 0.0006 |
| 1 | 85 (23%) | 16 (17%) | 69 (25%) |  |
| 2 | 253 (69%) | 65 (66%) | 188 (70%) |  |
| 3 | 29 (8%) | 16 (17%) | 13 (5%) |  |
| %VC, mean ± SD (%) | 102.5 ± 15.3 | 99.7 ± 15.5 | 103.4 ± 15.2 | 0.043 |
| FEV1/FVC ratio, mean ± SD (%) | 74.1 ± 9.1 | 70.6 ± 11.3 | 75.4 ± 7.9 | <0.0001 |
| Comorbidity |  |  |  |  |
| Diabetes mellitus | 48 (13%) | 15 (15%) | 33 (12%) | 0.48 |
| Respiratory disease | 140 (38%) | 56 (58%) | 84 (31%) | <0.0001 |
| Chronic obstructive pulmonary disease | 112 (31%) | 49 (51%) | 63 (23%) | <0.0001 |
| Cardiovascular disease | 170 (46%) | 52 (43%) | 128 (47%) | 0.55 |
| Clinical stage |  |  |  | 0.72 |
| 0, I | 94 (25%) | 22 (23%) | 72 (27%) |  |
| II | 68 (19%) | 19 (20%) | 49 (18%) |  |
| III | 137 (37%) | 40 (41%) | 97 (36%) |  |
| IV | 68 (19%) | 16 (16%) | 52 (19%) |  |
| Preoperative treatment |  |  |  | 0.48 |
| Absent | 162 (44%) | 46 (48%) | 116 (43%) |  |
| Neoadjuvant chemotherapy | 130 (36%) | 35 (36%) | 95 (35%) |  |
| Neoadjuvant chemoradiotherapy | 42 (11%) | 11 (11%) | 31 (12%) |  |
| Definitive chemoradiotherapy | 33 (9%) | 5 (5%) | 28 (10%) |  |
| *SD,* standard deviation*; VC*, vital capacity; *FEV*, forced expiratory volume; *FVC*, forced vital capacity | | | | |
| * Brinkman index was calculated as follows: number of cigarettes/day x smoking duration (year) | | | | |

Supplementary Digital Content Table 2. Characteristics of the patients who underwent minimally invasive esophagectomy based on the low-attenuation volume ratio

|  |  | Low attenuation volume ratio | | *p* |
| --- | --- | --- | --- | --- |
| Clinical, epidemiological, and surgical feature | Total *N* | High | Low |  |
| All cases | 409 | 122 | 287 |  |
| Age, mean ± SD | 66.1 ± 7.8 | 67.1 ± 7.6 | 65.7 ± 7.9 | 0.11 |
| Sex Male | 350 (86%) | 107 (88%) | 243 (85%) | 0.54 |
| Body mass index, mean ± SD (kg/m²) | 22.1 ± 3.1 | 20.9 ± 3.0 | 22.6 ± 3.1 | <0.0001 |
| *Brinkman Index, mean ± SD | 740 ± 530 | 940 ± 550 | 660 ± 500 | <0.0001 |
| Performance status |  |  |  | 0.98 |
| 0 | 385 (94%) | 115 (94%) | 270 (94%) |  |
| 1 | 21 (5%) | 6 (5%) | 15 (5%) |  |
| 2 | 3 (1%) | 1 (1%) | 2 (1%) |  |
| American Society of Anesthesiologists Physical Status |  |  |  | 0.11 |
| 1 | 72 (18%) | 16 (13%) | 56 (20%) |  |
| 2 | 320 (78%) | 98 (80%) | 222 (77%) |  |
| 3 | 17 (4%) | 8 (7%) | 9 (3%) |  |
| %VC, mean ± SD (%) | 101.9 ± 14.2 | 102.6 ± 14.2 | 101.6 ± 14.3 | 0.52 |
| FEV1/FVC ratio, mean ± SD (%) | 73.1 ± 8.6 | 70.0 ± 10.8 | 74.4 ± 7.0 | <0.0001 |
| Comorbidity |  |  |  |  |
| Diabetes mellitus | 102 (25%) | 23 (19%) | 79 (28%) | 0.080 |
| Respiratory disease | 128 (31%) | 58 (48%) | 70 (24%) | <0.0001 |
| Chronic obstructive pulmonary disease | 121 (30%) | 53 (43%) | 68 (24%) | 0.0001 |
| Cardiovascular disease | 230 (56%) | 72 (59%) | 158 (55%) | 0.51 |
| Clinical stage |  |  |  | 0.087 |
| 0, I | 212 (52%) | 52 (43%) | 160 (56%) |  |
| II | 73 (18%) | 27 (22%) | 46 (16%) |  |
| III | 94 (23%) | 31 (25%) | 63 (22%) |  |
| IV | 30 (7%) | 12 (10%) | 18 (6%) |  |
| Preoperative treatment |  |  |  | 0.10 |
| Absent | 252 (61%) | 67 (55%) | 185 (65%) |  |
| Neoadjuvant chemotherapy | 127 (31%) | 43 (35%) | 84 (29%) |  |
| Neoadjuvant chemoradiotherapy | 27 (7%) | 12 (10%) | 15 (5%) |  |
| Definitive chemoradiotherapy | 3 (1%) | 0 (0%) | 3 (1%) |  |
| *SD,* standard deviation*; VC*, vital capacity; *FEV*, forced expiratory volume; *FVC*, forced vital capacity | | | | |
| * Brinkman index was calculated as follows: number of cigarettes/day x smoking duration (year) | | | | |
